# Neuro–immune dysregulation after traumatic brain injury: immunomodulatory strategies to prevent infectious complications

**DOI:** 10.3389/fnmol.2026.1902464

**Published:** 2026-08-03

**Authors:** Sylvain Gourier, Etienne Botquelen, Olivier Langeron, Anaïs Caillard, Marwan Bouras

**Affiliations:** 1 Department of Anesthesiology and Critical Care Medicine, Brest University Hospital Centre, Brest, France; 2 Univ Brest, Laboratoire ORPHY EA 4324, Brest, France; 3 Univ Brest, INSERM, EFS, UMR 1078, GGB, Brest, France

**Keywords:** brain injury-induced immunosuppression, immune endotyping, immunomodulation, neuroinflammation, nosocomial infection, precision medicine, traumatic brain injury

## Abstract

Traumatic brain injury (TBI) induces a dynamic immune response that increases susceptibility to infectious complications, particularly ventilator-associated pneumonia, bloodstream infections, and central nervous system infections. These infections prolong mechanical ventilation and intensive care unit stay and may adversely affect neurological recovery. This vulnerability is driven by brain injury-induced immune suppression, characterized by a coexisting inflammatory response and a compensatory immunodepression, including lymphopenia, impaired antigen presentation, monocyte and neutrophil dysfunction, reduced cytokine responsiveness, and neuroendocrine dysregulation. Bidirectional neuro-immune interactions contribute to these alterations: damage-associated molecular patterns and microglial activation initiate neuroinflammation, while sympathetic hyperactivation and hypothalamic-pituitary-adrenal axis dysfunction promote peripheral immune impairment. Emerging pathways, including cGAS-STING signaling, may further link neuronal injury, neuroinflammation, and immune paralysis. This narrative review examines experimental and clinical evidence for targeted immunomodulatory strategies intended to reduce infectious complications after TBI while avoiding exacerbation of secondary brain injury. Candidate approaches include granulocyte-macrophage and granulocyte colony-stimulating factors, interferon-γ and interleukin-7, mesenchymal stromal cells, and neuromodulatory interventions targeting adrenergic or cholinergic pathways. Although several strategies have restored immune cell function or reduced infectious burden in preclinical models, clinical translation remains limited by small heterogeneous cohorts, variable timing of intervention, and insufficient assessment of infection-centered outcomes. A central challenge is that post-TBI immunity is dynamic and heterogeneous, ranging from hyperinflammatory to profoundly immunosuppressed profiles, while likely differing between the central nervous system and the peripheral/systemic immune compartments. Biomarker-guided identification of immune endotypes may therefore be essential to select appropriate interventions and therapeutic windows. Sex-related immune differences may also influence infection risk and treatment response through the immunomodulatory and barrier-protective effects of sex hormones. Future studies should combine serial immune profiling with clinically meaningful infectious and neurological outcomes. Precision immunomodulation, tailored to immune phenotype, timing, and potentially sex-hormone status, may provide a rational strategy to reduce infectious morbidity and improve outcomes after TBI.

## 1 Introduction

Traumatic brain injury (TBI) remains a major cause of death and long-term disability worldwide, particularly among young adults (Maas et al., 2022). In patients with moderate to severe TBI admitted to the intensive care unit (ICU), outcome is determined not only by the primary cerebral injury and subsequent intracranial complications, but also by systemic complications occurring during critical illness. Among these, hospital-acquired infections represent a major burden (Meisel et al., 2005; Prieto-Alvarado et al., 2025). Ventilator-associated pneumonia is particularly frequent in mechanically ventilated patients with severe TBI, with reported incidences of approximately 40%–50% (Robba et al., 2020). Bloodstream infections and central nervous system infections further contribute to morbidity in this vulnerable population (Sharma et al., 2019; Bouras et al., 2022). In addition, infection may aggravate secondary cerebral insults (Sharma et al., 2019).

The increased susceptibility of patients with TBI to infection cannot be explained solely by invasive devices, aspiration risk or prolonged mechanical ventilation. Severe brain injury rapidly alters systemic immune function (Bouras et al., 2022). Following the initial cerebral insult, blood–brain barrier disruption, release of damage-associated molecular patterns, and activation of microglia and astrocytes promote an early neuroinflammatory and systemic inflammatory response (Meisel et al., 2005; Kourbeti et al., 2012; Sharma et al., 2019). This early immune activation is initiated by the release of endogenous danger signals, including extracellular ATP, HMGB1, heat shock proteins and mitochondrial components, which activate pattern-recognition receptors on resident glial cells and infiltrating immune cells (Meisel et al., 2005; Vourc’h et al., 2018; Cáceres et al., 2024). In this context, TBI is increasingly understood not as an isolated focal neurological lesion, but as a systemic neuroimmune disorder in which intracranial sterile inflammation and peripheral immune dysfunction develop in parallel.

Although this response contributes to tissue repair and antimicrobial defense, excessive or prolonged inflammation may trigger compensatory immunoregulatory mechanisms. Through autonomic imbalance, hypothalamic–pituitary–adrenal axis activation and immune-cell reprogramming, TBI can induce a state of peripheral immune dysfunction characterized by lymphopenia, impaired monocyte antigen presentation, altered neutrophil and macrophage function, and reduced cytokine responsiveness (Meisel et al., 2005; Cáceres et al., 2024). This phenomenon has been conceptualized as central nervous system injury-induced immune deficiency syndrome, in which the injured brain suppresses peripheral immunity through neuroendocrine and autonomic pathways, thereby increasing vulnerability to nosocomial infections (Hazeldine et al., 2015).

This dynamic coexistence of neuroinflammation and systemic immunosuppression has major therapeutic implications. Broad anti-inflammatory therapy may worsen host defense, whereas immune stimulation may exacerbate secondary brain injury. Immunomodulatory strategies after TBI should therefore aim to restore immune competence selectively, according to the patient’s immune phenotype and the timing of intervention.

This narrative review examines the mechanisms linking TBI-induced immune dysfunction to infectious complications and summarizes current experimental and clinical evidence for targeted immunomodulatory strategies.

## 2 From cerebral injury to systemic immune vilnerability: a concise framework

**FIGURE 1:**
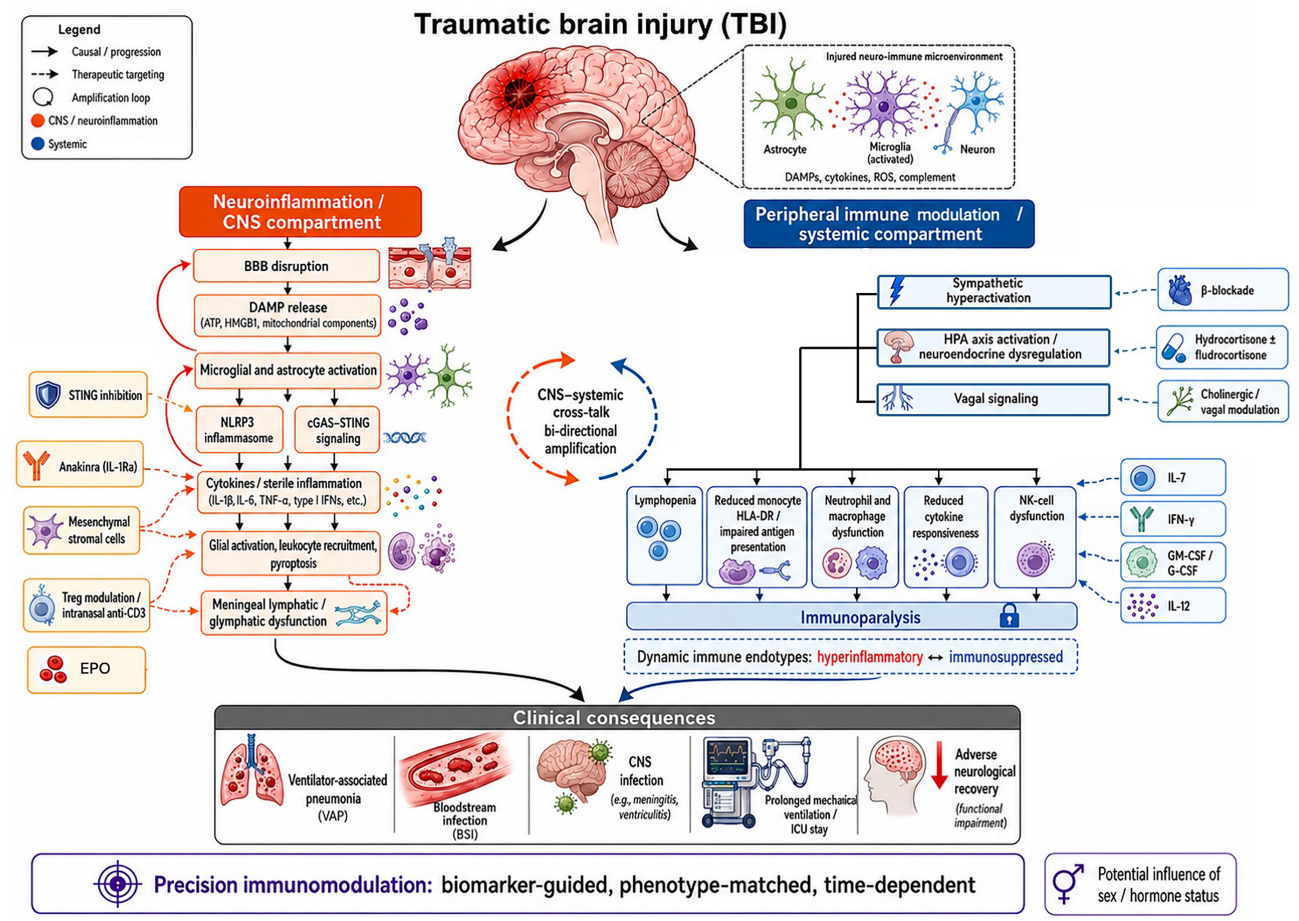
Pathophysiological mechanisms and therapeutic opportunities in traumatic brain injury–induced neuroimmune dysregulation. Overview of the pathophysiological mechanisms and potential therapeutic targets involved in neuroimmune dysregulation after traumatic brain injury. The initial brain insult promotes blood–brain barrier disruption, release of damage-associated molecular patterns, and activation of microglia and astrocytes, leading to central neuroinflammation through pathways such as the NLRP3 inflammasome and cGAS–STING signaling. In parallel, systemic neuroendocrine and autonomic responses, including sympathetic hyperactivation, hypothalamic–pituitary–adrenal axis dysregulation and altered vagal signaling, contribute to peripheral immune suppression. These converging processes may result in dynamic immune endotypes ranging from hyperinflammation to immunosuppression and immunoparalysis, thereby increasing the risk of infectious complications, prolonged intensive care support and impaired neurological recovery. The figure also summarizes emerging therapeutic strategies, including inflammasome or STING pathway inhibition, cytokine-targeted approaches, cellular therapies, autonomic modulation and immune-restorative interventions, supporting a precision immunomodulation framework guided by biomarkers, immune phenotype and timing after injury. Created with Servier Medical Art and BioRender.

The pathophysiological mechanisms are summarized in Figure 1.

### 2.1 Barrier failure and early neuroinflammation

The primary lesion occurs at the moment of impact and is followed by secondary biological injury that develops over hours to days (Jassam et al., 2017; Ware et al., 2022). Mechanical deformation, vascular injury and axonal damage impair the blood-brain barrier (BBB), exposing cerebral tissue to circulating proteins and permitting peripheral immune-cell recruitment (Alves, 2014). In human TBI, dynamic contrast-enhanced magnetic resonance imaging has demonstrated increased BBB permeability within and outside overt lesions, with variable persistence over time (Ware et al., 2022). Barrier disruption is therefore relevant not only as an acute structural event but also as a gateway for prolonged neuroimmune interaction (Cáceres et al., 2024).

Injured neurons, astrocytes, endothelial cells and axons release damage-associated molecular patterns, including extracellular ATP, high-mobility group box 1 and mitochondrial components (Jassam et al., 2017; Balança et al., 2021). These signals activate pattern-recognition receptors and inflammasome pathways in resident and infiltrating immune cells, promoting cytokine release, endothelial activation and pyroptotic cell death (Zhu et al., 2026). Among these pathways, NLRP3 inflammasome activation links DAMP sensing to caspase-1 activation, IL-1β/IL-18 maturation and gasdermin D-mediated pyroptosis, thereby amplifying sterile neuroinflammation after TBI (Hu et al., 2020; O’Brien et al., 2020; Cáceres et al., 2024). In parallel, the cytosolic DNA-sensing cGAS–STING pathway has emerged as another key regulator of post-traumatic neuroinflammation (Abdullah et al., 2018). After TBI, mitochondrial damage can release cytosolic DNA, activating cGAS–STING signaling and downstream TBK1–IRF3/NF-κB pathways, which induce type I interferons and inflammatory mediators.

Microglia, the resident macrophages of the CNS, represent the first line of immune defense in the acute phase of traumatic brain injury (TBI) as they rapidly adopt an activated phenotype limiting the spread of injury and initiating repair processes (Prinz et al., 2019; Liu et al., 2020). Astrocytes undergo a complex and dynamic transformation referred to as reactive astrogliosis, essential for maintaining cerebral homeostasis, synaptic activity and integrity of the blood–brain barrier (BBB). Microglia and astrocytes have traditionally been described as binary “pro-inflammatory” or “reparative” populations but contemporary transcriptomic studies show heterogeneous, time- and context-dependent states, a finding that argues against therapies designed around a binary classification (Fritsch et al., 2024; Jha et al., 2024). Brain clearance pathways may also influence persistence of inflammation (Bolte et al., 2020; Bouras et al., 2022). Meningeal lymphatic vessels provide a structural route linking central nervous system fluid and immune signals with cervical lymphatic drainage (Louveau et al., 2015). Experimental TBI disrupts meningeal lymphatic function and worsens injury-related outcomes, while BBB and glymphatic abnormalities may facilitate accumulation of inflammatory mediators and cellular debris (Bolte et al., 2020). The glymphatic system is a perivascular brain clearance pathway that enables cerebrospinal fluid–interstitial fluid exchange and removal of metabolic waste (Hablitz and Nedergaard, 2021; Ferrara et al., 2022). After TBI, glymphatic dysfunction may impair clearance of inflammatory mediators and injury-related proteins, promoting edema, persistence of neuroinflammation, and possibly long-term neurodegenerative processes (Ferrara et al., 2022; Hussain et al., 2023). These mechanisms are important for infection-focused immunomodulation because they illustrate why a systemic therapy could alter both host defense and intracranial inflammatory burden.

Post-traumatic inflammation is not confined to the acute phase. Human neuropathological data demonstrate persistent microglial activation and white-matter degeneration years after a single TBI, while positron-emission tomography studies have identified chronic microglial activation in living patients long after injury (Ramlackhansingh et al., 2011; Johnson et al., 2013). These observations support the concept of tertiary injury and are clinically relevant when evaluating immune-restorative therapies: any intervention intended to decrease infection risk must also be assessed for its effect on longer-term neuroinflammatory and functional outcomes.

### 2.2 From local inflammation to systemic immune suppression

Cerebral injury rapidly communicates with peripheral immunity through humoral, autonomic and endocrine routes (Meisel et al., 2005). An early systemic inflammatory response may coexist with a compensatory anti-inflammatory programme, classically described as the systemic inflammatory response syndrome (SIRS)/Compensatory anti-inflammatory response syndrome (CARS) imbalance. When the latter predominates, reduced monocyte HLA-DR expression, impaired antigen presentation and lymphocyte dysfunction may create an immunoparalytic phenotype (Hazeldine et al., 2015; Bouras et al., 2022). Sympathoadrenal activation, hypothalamic-pituitary-adrenal activation and vagal signaling can contribute to this shift. This framework provides a plausible biological basis for increased infection susceptibility after severe brain injury, while emphasizing that the intensity and duration of immune changes vary with age, extracranial trauma, transfusion, surgery and infection itself (Bouras et al., 2022).

The lung represents one of the most clinically relevant extracranial targets (Asehnoune et al., 2014). Severe TBI can alter pulmonary innate immunity, while ventilation, aspiration and reduced airway protection provide additional infectious risk (Asehnoune et al., 2018). Experimental studies demonstrate that post-TBI bacterial pneumonia can increase mortality and sustain defects in monocyte innate responses; other work supports sex-dependent differences in bacterial clearance (Pittet et al., 2021). More generally, inflammatory injury may reprogram alveolar macrophages and produce a long-lasting state of impaired local antibacterial function (Roquilly et al., 2020). These findings support an integrated “brain-lung axis” in which pulmonary infection is not solely a consequence of intubation and exposure, but may also reflect altered immune competence after brain injury (Mascia, 2009; Asehnoune et al., 2018; Huang et al., 2025; Mancabelli et al., 2026).

The intestine may contribute to this systemic amplification (Hanscom et al., 2021). Experimental TBI is associated with altered gut barrier function, dysbiosis and bidirectional inflammatory signaling between the intestine and the injured brain (Ma E. L. et al., 2017). Although the relationship between gut alterations and nosocomial infection in human TBI remains insufficiently defined, the brain-gut axis provides a further mechanism through which autonomic imbalance, barrier impairment and microbial products could influence systemic immunity (Hanscom et al., 2021). This pathway is therapeutically attractive but currently less mature than the brain-lung axis for infection-prevention trials (Hanscom et al., 2021).

## 3 Infectious complications after TBI: the clinical problem that immunomodulation must address

Mechanically ventilated patients with severe TBI are at particularly high risk of VAP. In a large multicenter prospective observational cohort, VAP was common among patients with TBI receiving ventilation and was associated with increased morbidity (Robba et al., 2020). Earlier studies have similarly reported substantial rates of early-onset VAP and relationships with clinically important outcomes, including cerebral oxygenation and length of intensive care (Esnault et al., 2017; Li et al., 2020). A recent meta-analysis confirmed that VAP in TBI is associated with prolonged ventilation, intensive care stay and hospital stay, even when its relationship with mortality is less consistent across studies (Prieto-Alvarado et al., 2025).

Infection prevention after acute brain injury already includes interventions with evidence distinct from immunotherapy. For example, a recent randomized trial showed that a single early dose of ceftriaxone reduced early VAP in mechanically ventilated patients with acute brain injury (Dahyot-Fizelier et al., 2024). Such approaches are clinically important, but antibiotic prophylaxis and immune restoration address different biological questions. Antimicrobial reduce early bacterial infection risk but cannot be prolonged for ecological considerations, so immunomodulation must follow to correct host-response and avoid recurrent infections (Bouras et al., 2022). Antibiotic prophylaxis targets early microbial exposure, whereas immunomodulation targets persistent host-response failure.

A key limitation of the current literature is that many mechanistic studies focus on inflammatory biomarkers or neurological outcomes, whereas clinical studies of infection rarely include serial immune phenotyping (Samanta et al., 2024). This disconnect makes it difficult to identify which infections are linked to biological immunosuppression and which result principally from clinical exposures. A therapeutic trial should not assume that all severe TBI patients have the same immune defect. It should instead identify an actionable phenotype and test whether its correction reduces infection without worsening brain injury (Gandasasmita et al., 2024). It is essential to individualize therapeutic strategies according to the immune phenotype. Indeed, this phenotype exhibits both inter-individual variability and dynamic intra-individual changes over time.

The distinction between early and late infection may also be biologically meaningful. Early pneumonia may be strongly influenced by aspiration, emergency airway management and initial ventilation, whereas later or recurrent infection may provide a better window in which persistent immune dysfunction contributes to risk (Esnault et al., 2017; Doran et al., 2020; Robba et al., 2020). For this reason, future immune-directed trials should not use a single undifferentiated “pneumonia” outcome: early VAP, late VAP, recurrent infection and bloodstream infection should be predefined separately. This distinction is particularly important for immunomodulation: immune-restorative interventions may be more relevant for late or recurrent infections associated with persistent monocyte/macrophage dysfunction than for immediate aspiration-driven pneumonia.

## 4 A precision approach: immune endotypes and biomarker-guided therapy

A biomarker-guided strategy is supported indirectly by precision-immunotherapy studies in sepsis (Leventogiannis et al., 2022; Giamarellos-Bourboulis et al., 2026). The PROVIDE trial introduced a phenotype-matched approach using anakinra for a macrophage activation-like syndrome phenotype and recombinant interferon-gamma (IFN-γ) for immunoparalysis (Leventogiannis et al., 2022). More recently, the ImmunoSep randomized clinical trial applied the same biological logic in patients with sepsis, pneumonia or bacteremia: precision immunotherapy achieved the prespecified improvement in organ dysfunction by day 9 more often than placebo (Giamarellos-Bourboulis et al., 2026).

These findings do not demonstrate efficacy in TBI, where blood-brain barrier disruption, neuroinflammation and cerebral perfusion constraints generate a distinct safety problem. They do, however, provide a clinically mature methodological template: serial immune profiling could identify patients with sustained monocyte deactivation, hyperinflammatory profiles or severe lymphocyte dysfunction, in whom a targeted intervention might be evaluated while monitoring infections and neurological outcomes simultaneously (Sadek et al., 2026).

Research in immunomodulation following traumatic brain injury should draw on advances made in sepsis research, where therapeutic strategies are increasingly guided by patient stratification based on endotypes and biomarkers.

Indeed, the concept of immune endotypes is increasingly relevant in TBI (Bouras et al., 2022; Samanta et al., 2024). A multicenter analysis using inflammatory mediators from CENTER-TBI and TRACK-TBI cohorts identified immune-response endotypes associated with global outcome, supporting the view that systemic inflammatory responses vary meaningfully between patients (Samanta et al., 2024). Such work does not yet define a bedside indication for immune therapy, but it challenges the traditional approach of enrolling unselected TBI populations in interventional trials. A treatment designed to restore immune competence is unlikely to benefit patients whose predominant problem is uncontrolled early inflammation, and an anti-inflammatory treatment may be inadequate in patients already exhibiting immunoparalysis (Magatti et al., 2023). Thus, immunomodulation after TBI should be phenotype-matched: anti-inflammatory approaches for selected hyperinflammatory profiles, immune-restorative strategies for immunoparalysis, and avoidance of either approach in patients without a corresponding biological signal.

Regarding biomarkers, Monocyte Human Leukocyte Antigen (HLA) -DR expression has been extensively used in critical illness as a measure of impaired antigen-presentation capacity (Lukaszewicz et al., 2009; Monneret and Venet, 2014). An observational study in young and elderly patients with traumatic brain injury reported reduced HLA-DR expression, suggesting impaired antigen presentation capacity (Magatti et al., 2023). Absolute lymphocyte counts, CD4/CD8 subsets, lymphocyte and neutrophil dysfunction may identify immune impairment (Conway Morris et al., 2013). In TBI, these measures should be interpreted alongside injury severity, extracranial trauma, corticosteroid or catecholamine exposure, microbiological status and time since injury.

Timing should be incorporated into the definition of an endotype. An admission cytokine surge may reflect lesion severity and sterile inflammation rather than immune competence, whereas a persistently low functional response several days later may identify a patient unable to clear bacteria effectively (Magatti et al., 2023; Samanta et al., 2024). Combining a functional assay with a cellular marker may also be safer than relying on cytokine concentration alone, because circulating inflammatory mediators can be elevated while antigen presentation or antimicrobial cell function is impaired (Meisel et al., 2005; Magatti et al., 2023).

## 5 Candidate immunomodulatory interventions

Candidate immunomodulatory interventions are summarized in Table 1 and shown in Figure 1.

**TABLE 1:** Candidate immunomodulatory interventions for prevention of infectious complications after traumatic brain injury.

Intervention/strategy	Main immune target	Rationale after TBI	Evidence relevant to infection prevention	Potential target population
G-CSF	Neutrophil production and antibacterial function	May increase neutrophil counts and enhance innate antibacterial defense.	Phase II severe brain-injury trial showed dose-dependent neutrophilia but no reduction in pneumonia or urinary tract infection; possible reduction in bacteremia.	Patients with documented neutropenia or impaired neutrophil function.
GM-CSF	Monocytes, macrophages, antigen presentation, neutrophil function	May reverse monocyte deactivation and restore myeloid antibacterial competence.	Preclinical TBI/polytrauma and pneumonia models showed improved immune-cell responses and reduced lung bacterial load without worsening neuroinflammation.	Persistent monocyte deactivation, low HLA-DR, impaired phagocytosis, late/recurrent infections.
Interferon-γ	Monocyte/macrophage activation and HLA-DR expression	May restore antigen presentation in immunoparalysis.	Evidence mainly from sepsis/critical illness; no direct TBI efficacy data; safety concerns with non-selected use.	Documented peripheral immunoparalysis with low HLA-DR and recurrent/late infection.
Interleukin-7	T-cell survival and expansion	May correct profound post-injury lymphopenia and restore adaptive immunity.	Septic shock data show increased CD4/CD8 counts; no TBI infection-prevention data.	Persistent severe lymphopenia after TBI.
Interleukin-12	NK-cell IFN-γ production and cytotoxicity	May reverse post-TBI NK-cell dysfunction.	*Ex vivo* human TBI data suggest restoration of NK-cell IFN-γ production and cytotoxic capacity.	Patients with documented NK-cell dysfunction.
IL-1 receptor antagonism/anakinra	IL-1-mediated neuroinflammation	May attenuate early sterile neuroinflammation and protect BBB integrity.	Experimental and early clinical safety rationale; no evidence of reduced infections after TBI.	Hyperinflammatory endotype rather than immunoparalysis.
Corticosteroid-axis modulation	Stress-response dysregulation, NK cells, dendritic-cell survival	May modulate maladaptive neuroendocrine–immune responses.	Corti-TC did not reduce pneumonia overall; *post hoc* data suggest possible benefit in high cortisol/CRP subgroup.	Selected patients with stress-axis dysregulation, e.g., high cortisol/CRP ratio.
Erythropoietin	Regulatory T cells, cytokine balance, neuroinflammation	Pleiotropic endocrine-related agent with neuroprotective and immunomodulatory effects.	Experimental models show increased Tregs and anti-inflammatory shift; clinical infection prevention not demonstrated.	Unclear; potentially selected inflammatory/immunoregulatory phenotypes.
Beta-blockade	Sympathetic overactivation, T-cell exhaustion, pulmonary innate defense	May counteract catecholamine-driven immune dysfunction after TBI.	Clinical trials suggest mortality benefit and shorter ventilation, but were not designed to test infection prevention; experimental data support restoration of T-cell function.	Patients with marked sympathetic activation and immune dysfunction.
Vagal/cholinergic modulation	Cholinergic anti-inflammatory pathway	May attenuate excessive inflammation and preserve barrier integrity.	Experimental rationale only; reduced inflammation could also impair antimicrobial defense.	Selected hyperinflammatory profiles.
cGAS–STING inhibition	Sterile neuroinflammation, type I interferon/NF-κB signaling	May reduce DNA-sensing-driven neuroinflammation after cellular injury.	Preclinical neuroprotective data; not established as infection-prevention therapy.	Hyperinflammatory neuroinflammatory phenotype.
Mesenchymal stromal cells	Cytokine signaling, glial activation, immune homeostasis	May modulate neuroinflammation and promote repair.	Preclinical support and ongoing translational programmes; no human evidence of fewer infections.	Selected patients with persistent neuroinflammation or immune dysregulation.
Sex- and hormone-informed strategies	Sex-specific immune responses, estrogen/progesterone pathways	Sex and hormonal status may influence bacterial clearance and response to immunomodulation.	Experimental post-TBI pneumonia data suggest sex-dependent bacterial clearance and estrogen effects; progesterone phase III trials failed for neurological benefit and did not assess infection endpoints.	Future trials stratified by sex, age, and hormonal status.

BBB, blood–brain barrier; BSI, bloodstream infection; G-CSF, granulocyte colony-stimulating factor; GM-CSF, granulocyte–macrophage colony-stimulating factor; HLA-DR, human leukocyte antigen–DR; IFN, interferon; IL, interleukin; NK, natural killer; TBI, traumatic brain injury; Tregs, regulatory T cells; VAP, ventilator-associated pneumonia.

### 5.1 Restoring myeloid antibacterial function: G-CSF and GM-CSF

Granulocyte colony-stimulating factor (G-CSF) is attractive because it increases neutrophil production and may enhance antibacterial functions (Heard et al., 1998). It is also one of the few immunostimulatory therapies previously tested directly in acute severe brain-injury populations (Heard et al., 1998). In a multicenter, double-blind, placebo-controlled phase II study including patients with acute TBI or cerebral hemorrhage, filgrastim produced a dose-dependent neutrophilia but did not reduce pneumonia or urinary tract infection; a reduction in bacteremia was observed. This study establishes feasibility but does not support routine prophylactic G-CSF use, particularly because its cohort was small and not selected according to neutrophil dysfunction. Granulocyte-macrophage colony-stimulating factor (GM-CSF) may be more relevant to post-TBI immunoparalysis because it acts on monocytes, macrophages and antigen-presenting functions in addition to granulopoiesis (Bahader et al., 2025). In a juvenile rat model combining TBI and hemorrhagic polytrauma, systemic GM-CSF improved peripheral immune-cell responses without evidence of increased cerebral pro-inflammatory cytokines or worsened function (Sribnick et al., 2024). In a subsequent model incorporating lung bacterial challenge, GM-CSF increased MHC-II-high monocytes and splenic CD3-positive cells and reduced lung bacterial load without worsening glial activation, neuronal loss or memory dysfunction (Bahader et al., 2025). These are important proof-of-concept data because they directly link immune restoration to infection-relevant endpoints after TBI, although they remain preclinical. A clinical GM-CSF trial should therefore be targeted rather than prophylactic for all patients.

In this perspective, neutrophil function deserves greater emphasis: impaired neutrophil phagocytosis has been linked to nosocomial infection after TBI (Magatti et al., 2023), while studies in sepsis and broader critical illness suggest that GM-CSF may restore monocyte HLA-DR expression and improve defective neutrophil phagocytosis (Lukaszewicz et al., 2009; Monneret and Venet, 2014). These findings support integrating functional neutrophil assays, rather than cell counts alone, into future TBI trial eligibility criteria and pharmacodynamic endpoints.

### 5.2 Cytokine-based immune restoration after TBI

1. Interferon-gamma enhances macrophage activation, major histocompatibility complex expression and intracellular pathogen clearance (Döcke et al., 1997; Nakos et al., 2002). TBI can induce an immunoparalytic phenotype characterized by impaired monocyte antigen presentation, notably through reduced HLA-DR expression (Hazeldine et al., 2015). IFN-γ is therefore attractive because it can restore monocyte HLA-DR expression and improve antigen-presenting capacity, a mechanism already explored in trauma and sepsis (Livingston, 1994; Giamarellos-Bourboulis et al., 2026). The PROVIDE trial and ImmunoSep supported a precision-immunotherapy strategy in sepsis, with treatment allocated according to immune phenotype rather than given indiscriminately (Leventogiannis et al., 2022; Giamarellos-Bourboulis et al., 2026). In a multicenter randomized placebo-controlled trial, IFN-γ-1b was evaluated for the prevention of hospital-acquired pneumonia in mechanically ventilated critically ill patients with acute organ failure, among whom neurological failure, defined as coma, was reported in 83.5% of cases (Roquilly et al., 2023). The trial was stopped early after interim safety analysis because of a potential signal of harm. IFN-γ-1b did not significantly reduce the composite outcome of hospital-acquired pneumonia or death at day 28 compared with placebo, and serious adverse events were numerically more frequent in the treatment group. Exploratory analyses suggested that patients with a reduced CCL17 response to IFN-γ may represent a subgroup at higher risk of pneumonia, highlighting the need for biomarker-guided rather than systematic immune stimulation. In selected TBI patients with persistent monocyte deactivation, low HLA-DR and recurrent or late infections, IFN-γ could be evaluated as a targeted immune-restorative therapy, provided that neurological safety and intracranial inflammation are closely monitored. In septic patients with monocyte deactivation, IFN-gamma restored monocyte HLA-DR expression and immune responsiveness, establishing a mechanism for biomarker-directed immune restoration (Leventogiannis et al., 2022). However, evidence in patients with TBI is absent or indirect.
2. Interleukin-7 (IL-7) targets a different defect: profound and persistent lymphopenia. IL-7 is essential for T-cell survival and expansion (Mackall et al., 2011). The IRIS-7 randomized trial in septic shock demonstrated that recombinant human IL-7 increased circulating CD4 and CD8 lymphocyte counts in severely lymphopenic patients (Francois et al., 2018). Extrapolation to TBI is biologically plausible because adaptive immune alterations and lymphopenia can occur after severe injury, but the therapeutic balance is uncertain: T cells can participate in host defense as well as neuroinflammatory injury (Mazzeo et al., 2006; Dong et al., 2016). A TBI trial of IL-7 would therefore require sustained lymphopenia as an entry criterion and close monitoring for neurological and inflammatory safety signals.
3. Interleukin-12 (IL-12) is a new approach. In a study using blood samples from patients with TBI, Natural Killer (NK) cells displayed a differentiated phenotype and impaired responses to HLA class I–deficient targets, with reduced degranulation and IFN-γ production despite preserved antibody-dependent cellular cytotoxicity (Roquilly et al., 2017). IL-12 restored both IFN-γ production and NK-cell cytotoxic capacity *in vitro*, suggesting that IL-12 may partially reverse post-TBI NK-cell dysfunction and could represent an immune-restorative strategy to reduce infection susceptibility.
4. Interleukin-1 is an early mediator of post-traumatic neuroinflammation, and interleukin-1 receptor antagonism with anakinra has a strong experimental rationale and an emerging clinical safety framework (Lindblad et al., 2023; To et al., 2023). In patients with acute TBI, higher circulating IL-1 receptor antagonist concentrations have also been associated with better BBB integrity in the primary lesion region (To et al., 2023). Nevertheless, clinical evidence does not yet show that IL-1 blockade reduces disability or infectious complications after TBI.

Together, IFN-γ, IL-7, IL-12, and IL-1 receptor antagonism illustrate why cytokine-based immunomodulation after TBI should not be considered a single therapeutic strategy. These interventions target distinct immune defects, including monocyte deactivation, persistent lymphopenia, NK-cell dysfunction, and excessive IL-1–mediated neuroinflammation. Future trials should therefore avoid testing immunotherapies in unselected populations using generic infection endpoints alone. Instead, biomarker-guided platform or adaptive designs could allocate IFN-γ or GM-CSF to patients with monocyte deactivation, IL-7 to those with persistent lymphopenia, and potentially IL-12 to patients with documented NK-cell dysfunction, while evaluating IL-1 receptor antagonism in patients with evidence of excessive neuroinflammation.

Other candidate approaches, including TNF-alpha blockade (Tuttolomondo et al., 2014), neurokinin-1 receptor antagonism (Li et al., 2019) and immunometabolism modulators (Bouras et al., 2022), are mainly designed to target neuroinflammation, cerebral edema, or neuroprotection. Regulatory T-cell modulation has also emerged as an innovative preclinical strategy (Izzy et al., 2025). In a mouse model of contusional TBI, intranasal anti-CD3 therapy increased IL-10-producing regulatory T cells, reduced maladaptive microglial inflammation, enhanced microglial phagocytosis and improved neurological injury measures (Izzy et al., 2025). This approach is particularly relevant to the chronic neuroinflammatory component of TBI, but its consequences for antimicrobial host defense have not been established. Although experimental findings are biologically informative, none currently provides clinical evidence for the prevention of ICU-acquired infection after acute TBI.

### 5.3 Endocrine modulation

Severe TBI frequently involves dysregulation of the stress response, and glucocorticoid pathways influence both inflammation and antimicrobial immunity (Van De Garde et al., 2014; Cain and Cidlowski, 2017). In the Corti-TC multicenter randomized trial, hydrocortisone plus fludrocortisone did not significantly reduce hospital-acquired pneumonia or improve outcome overall in severe TBI (Asehnoune et al., 2014). A *post hoc* biomarker analysis of the Corti-TC cohort provides a more targeted hypothesis (Bouras et al., 2019). In patients with available admission samples, a total cortisol/CRP ratio greater than 3 was independently associated with hospital-acquired pneumonia; within this high-ratio subgroup, corticosteroid treatment was associated with a lower occurrence of pneumonia. Mechanistically, this effect may not be purely anti-inflammatory: experimental trauma-hemorrhage data showed that hydrocortisone limits post-traumatic immunosuppression by reducing IL-10-producing NK cells, thereby preserving dendritic-cell survival and maturation (Roquilly et al., 2014). Overall, these data suggest that studies targeting the corticotropic axis in traumatic brain injury should distinguish between the role of immunomodulation in preventing systemic infections and its role in limiting their impact on the central nervous system, while also considering the potential effects of these treatments on brain injury.

Beyond classical endocrine pathways, erythropoietin (EPO) represents another endocrine-related immunomodulatory candidate after TBI (Begemann et al., 2020; Alrasheed et al., 2024). EPO is a pleiotropic agent with neuroprotective and immunomodulatory properties that has been explored after TBI (Alrasheed et al., 2024). In experimental models, EPO reduced brain edema and modulated post-traumatic inflammation by increasing regulatory T cells in peripheral lymphoid organs and injured brain tissue, limiting immune-cell infiltration/activation, and shifting cytokine expression toward a more anti-inflammatory profile (Anderson et al., 2013; Zhou et al., 2017). However, clinical evidence remains inconsistent: meta-analyses suggest possible mortality benefit in some analyses, without clear improvement in neurological recovery or major safety concerns, but infection prevention has not been specifically demonstrated (Begemann et al., 2020; Alrasheed et al., 2024). Thus, EPO remains a biologically plausible but unproven candidate for preventing post-TBI infections.

### 5.4 Autonomic modulation: beta-blockade and vagal pathways

Traumatic brain injury is frequently accompanied by sympathetic hyperactivation, with excessive catecholamine exposure contributing to metabolic stress, hemodynamic instability and immune dysregulation (Meisel et al., 2005; Loftus et al., 2016; Rizoli et al., 2017). Adrenergic signaling may impair lymphocyte function and pulmonary innate defense, providing a mechanistic rationale for beta-blockade as a potential immunomodulatory approach (Panina-Bordignon et al., 1997; Bellinger et al., 2008). Earlier syntheses, which largely included observational data, suggested an association between beta-blocker exposure and reduced mortality after TBI but remained vulnerable to confounding and did not primarily assess infectious outcomes (Hart et al., 2023). A recent systematic review and meta-analysis, restricted to randomized controlled trials in adults with acute TBI, provides a more precise understanding of the available evidence. Across seven trials, beta-blockers did not significantly reduce unfavorable long-term neurological outcome, but were associated with lower mortality and shorter mechanical-ventilation duration (Bouras et al., 2025). Furthermore, experimental data provide a biological rationale for a potential immune-modulatory effect. In a rat model of acute TBI, sympathetic activation was associated with increased circulating norepinephrine, upregulation of PD-1 on CD4^+^ and CD8^+^ T cells, and impaired T-cell production of IFN-γ and TNF-α. Propranolol reversed these abnormalities *in vivo*, while norepinephrine reproduced them *in vitro*, and PD-1 blockade restored T-cell function. Thus, β-blockade may partially counteract SNS-driven T-cell exhaustion after TBI, although its clinical benefit currently appears better supported for survival than for infection prevention (Yang et al., 2019). By attenuating excessive adrenergic signaling, beta-blockers might preserve cellular immune function and pulmonary antibacterial activity (Loftus et al., 2016); however, existing clinical trials were not designed to demonstrate immune restoration or prevention of VAP. Future studies should prospectively measure pneumonia and other infection, monocyte HLA-DR, cytokine responsiveness and catecholamine exposure.

Vagal pathways represent a complementary but earlier-stage neuromodulatory avenue (Lopez et al., 2012). Through the cholinergic anti-inflammatory pathway, vagal signaling can attenuate cytokine release and may preserve barrier integrity after experimental TBI (Lopez et al., 2012). Yet this dual action is precisely the safety challenge in infection prevention: reduced cerebral or systemic inflammation may be beneficial, while excessive anti-inflammatory signaling could further impair antimicrobial defense (Tang et al., 2020; Bouras et al., 2022). Neuromodulation should therefore remain an experimental strategy evaluated with both neuroinflammatory and infectious endpoints.

### 5.5 Molecular and cell-based approaches: cGAS-STING and mesenchymal stromal cells

The cyclic GMP-AMP synthase-stimulator of interferon genes (cGAS-STING) pathway is an increasingly compelling mechanistic bridge between cellular injury and neuroinflammation (Abdullah et al., 2018; Zhang et al., 2022). After TBI, double-stranded DNA released by injured cells may activate STING-dependent type I interferon and NF-kB signaling in microglia and astrocytes (Abdullah et al., 2018; Fritsch et al., 2024). Preclinical studies have associated STING signaling with detrimental neuroinflammation and NLRP3-related pyroptosis, while pharmacological STING inhibition with a small-molecule inhibitor reduced inflammatory gene expression and tissue damage in an experimental TBI model (Fritsch et al., 2024; Fryer et al., 2024). These data identify STING as a promising neuroprotective target, but not yet as an infection-prevention intervention: the same pathway contributes to antimicrobial defense, making infectious safety a central requirement for translation.

Mesenchymal stromal cells (MSCs) are another immunoregulatory approach with clinically relevant development (Pischiutta et al., 2021). Preclinical TBI studies suggest that MSCs modulate cytokine signaling, glial activation and repair processes, and meta-analytic evidence supports continued translational evaluation (Pischiutta et al., 2021; Sigaut et al., 2024). In addition to the MATRIx programme using bone-marrow-derived cells, the TRAUMACELL multicenter randomized controlled trial will evaluate repeated intravenous administration of Wharton’s jelly-derived MSCs in severe TBI, with post-traumatic neuroinflammation measured objectively by PET-MRI in the corpus callosum at 6 months (Zanier et al., 2023; Sigaut et al., 2024).

This rationale should be distinguished from neural stem/progenitor cell strategies aimed primarily at cell replacement or trophic support. MSCs are most relevant here because their proposed effects are immunoregulatory; however, no human TBI study has yet demonstrated improved antimicrobial competence or fewer infections after MSC administration. In an immunosuppressed patient, an intervention that shifts inflammatory signaling could either restore immune homeostasis or further reduce effective host defense. Trials of MSCs and other neuroinflammation-modulating approaches should therefore prospectively record VAP, bloodstream infection, antimicrobial exposure and immune biomarkers, alongside neurological imaging and functional outcomes.

### 5.6 Sex-informed and hormone-informed strategies

Sex may modify infection susceptibility and response to immunomodulation after brain injury (Pittet et al., 2021; Gourier et al., 2026). Sex-related differences may influence immune responses after injury/stress (Gourier et al., 2025): Females generally show stronger innate and adaptive immune responses (Bouman et al., 2005; Hannah et al., 2008), although these may vary with the menstrual cycle (Yovel et al., 2001). Compared with males, females tend to have higher phagocytic activity, stronger cytokine responses, and greater humoral immunity, including higher immunoglobulin levels. In contrast, males may have more NK cells, but these cells appear less functionally active (Yovel et al., 2001; Abdullah et al., 2012). In an experimental post-TBI pneumonia model, male mice showed worse bacterial clearance and survival than females, while estrogen treatment attenuated sex-dependent differences (Pittet et al., 2021). These findings are hypothesis-generating rather than clinically actionable, but they indicate that sex and hormonal status should not be ignored in future immune-intervention trials. This hormonal hypothesis has also led some authors to investigate progesterone as a potential neuroprotective therapy after TBI. However, two large phase III randomized trials published in 2014, ProTECT III and SYNAPSE, failed to show a clinical benefit (functional outcome, mortality, temperature) of progesterone in acute or severe TBI (Skolnick et al., 2014; Wright et al., 2014; Ma J. et al., 2017). However, infectious complications were not evaluated as clinical outcomes in these studies.

## 6 Toward precision immunomodulation trials in neurocritical care

The central translational obstacle is heterogeneity of TBI. Severe TBI is a diagnosis encompassing isolated trauma and multiple trauma, diffuse and focal lesions, different ages, varying exposure to blood products or surgery, and different durations of ventilation and sedation (Maas et al., 2022). Future studies should therefore enroll a clinically defined at-risk population and perform serial immune measurements, rather than relying on a single admission sample while keeping the design simple to ensure feasibility in clinical practice. An initial observational phase could define immune trajectories associated with infection (Robba et al., 2020; Magatti et al., 2023; Samanta et al., 2024), after which an interventional trial could enroll only patients meeting a prespecified biological criterion. Future trials should avoid universal immunostimulation in unselected TBI populations. Instead, immune profiling should be used to identify patients with persistent and actionable immune dysfunction, and each intervention should be matched to its intended biological target. Neurological safety must remain central, with careful exclusion or monitoring of patients at risk of worsening intracranial inflammation. Such an approach would improve biological plausibility while reducing unnecessary exposure to immunomodulatory therapies.

Finally, immune-targeted therapy should be integrated into, not substituted for, rigorous infection prevention and high-quality neurocritical care. VAP prevention bundles, careful airway management, early enteral strategies, antimicrobial stewardship and evidence-based prophylactic approaches remain the clinical foundation (Roquilly et al., 2015; Asehnoune et al., 2018; Dahyot-Fizelier et al., 2024). Immunomodulation may eventually offer additional benefit for a selected subgroup whose biological immune dysfunction persists despite standard care. The most credible pathway to translation is therefore not a universal treatment, but a staged and monitored intervention that targets a measured defect at a clinically appropriate time.

Translation will likely require an iterative rather than linear approach. Future studies must first determine which immune abnormalities are reproducible, measurable at the bedside, and genuinely associated with late or recurrent infections after TBI. Early trials should prioritize target engagement and neurological safety before moving to larger studies powered for infection-related outcomes. This pragmatic pathway would avoid premature efficacy testing while preserving the central objective: matching immunomodulation to a documented immune defect without increasing secondary brain injury.

## 7 Conclusion

Traumatic brain injury induces a complex and time-dependent immune disorder in which neuroinflammation and systemic immunosuppression may coexist. This duality explains why immunomodulation cannot currently be recommended as routine infection-prevention therapy after TBI. Overall, immunomodulation should not be viewed as a single therapeutic strategy: some interventions aim to restore impaired host defense, whereas others primarily target neuroinflammation or neuroendocrine dysregulation. Future research should therefore move beyond unselected populations and generic infection endpoints. The most credible path forward is to match immunomodulatory interventions to immune phenotype and timing, while assessing both infectious outcomes and neurological safety. This precision approach may ultimately help reduce infectious morbidity after TBI without increasing the risk of secondary brain injury.
